# Meta-analysis of risk factors for posttraumatic stress disorder in myocardial infarction

**DOI:** 10.1097/MD.0000000000036601

**Published:** 2024-01-19

**Authors:** Jingyu Liu, Lingyu Wang, Yimu Wang, Haiyan Fang, Xiang Wang

**Affiliations:** a College of Nursing, Anhui University of Chinese Medicine, Hefei, China.

**Keywords:** meta-analysis, myocardial infarction, posttraumatic stress disorder, risk factors

## Abstract

**Background:**

The aim of this study was to identify the risk factors for posttraumatic stress disorder in patients with myocardial infarction.

**Methods:**

Cohort, case-control, and cross-sectional studies on posttraumatic stress disorder (PTSD) in patients with myocardial infarction were searched from PubMed, Embase, Cochrane Library, Web of Science, China Biomedical Literature Database, China National Knowledge Infrastructure, Wanfang Data Knowledge Service Platform, and Technology Journal database. The Newcastle-Ottawa Quality Assessment Scale was used to score the quality of the included literature in the cohort and case-control studies, and the cross-sectional studies were scored using the American Agency for Health Care Quality and Research cross-sectional study quality evaluation criteria. The literature was screened independently by 2 researchers, and if there was no consensus, the inclusion was decided by a third party. The extraction content included first author, publication year, sample size, PTSD assessment tool, PTSD assessment time, PTSD incidence, influencing factors, and study type. Meta-analysis of data was performed using Stata17.0 software.

**Results:**

Ten studies were included, including 2 cohort studies, 7 cross-sectional studies, and 1 case-control study, with a total sample size of 2371 patients, including 26 influencing factors. The results of meta-analysis showed that the prevalence of PTSD in patients with myocardial infarction was 21.2%. Statistically significant influencing factors were gender (odd ratio [OR] = 3.124), neuroticism score (OR = 2.069), and age (OR = 0.913).

**Conclusions:**

The prevalence of PTSD in patients with myocardial infarction in China is higher than that in other countries. Female and neurotic personality are risk factors for developing PTSD in patients with myocardial infarction, and old age is protective factor for developing PTSD in patients with myocardial infarction. Targeted measures should be taken to prevent and reduce the occurrence and development of PTSD in patients with myocardial infarction in the future.

## 1. Introduction

Posttraumatic stress disorder (PTSD) is a mental disorder induced by a threatening or catastrophic stressful situation or event, characterized by intrusion into traumatic experiences, avoidance of traumatic events, and a persistent state of heightened alertness.^[1]^ PTSD has been confirmed by a large number of survivors of traumatic events such as catastrophic accidents and war, with an incidence of 10% to 20%.^[2]^ Its harm mainly lies in the psychological disorders of patients and the reduction of treatment compliance, which will affect the prognosis of patients. Myocardial infarction is one of the most serious types of coronary heart disease. Because of its rapid onset, rapid progression and high fatality rate, patients who survive the disease are prone to have serious psychological effects.^[3]^ As a major stressful traumatic event, the huge psychological stimulation usually causes patients to have stress disorders, affecting their physical and mental health and increasing the risk of cardiovascular adverse events.^[4]^ Studies have shown that the incidence of PTSD in patients with myocardial infarction is as high as 22%. To prevent the occurrence of PTSD in patients with myocardial infarction, the first step is to identify risk factors. At present, researchers have actively explored the risk factors of PTSD in patients with myocardial infarction, but the risk factors analyzed in various studies are different, and some research results are controversial. Therefore, meta-analysis was used in this study to integrate evidence of risk factors for PTSD in patients with myocardial infarction, aiming to provide references for clinical preventive management, so as to reduce the occurrence of PTSD in patients with myocardial infarction.

## 2. Methods

### 2.1. Study registration

This study was registered in the international prospective register of systematic reviews (PROSPERO registration number CRD42023443755) and was conducted following the Preferred Reporting Items for Systematic Reviews and Meta-Analyses (PRISMA) statement. This article reports the results of a literature search and does not involve any animal, cell, or human experimental research. This study did not require ethics approval in China.

### 2.2. Search strategy

We searched China National Knowledge Infrastructure, Technology Journal database, Wanfang Data Knowledge Service Platform, China Biomedical Literature Database, PubMed, Embase, Cochrane Library, and Web of Science databases. The search timeframe is from the creation of the database to July 2, 2023. The search method is the combination of subject words and free words. Chinese search terms were (“xinjigengsi” OR “xingeng” OR “xinjigengsai”) AND (“chuangshanghouyingjizhangai” OR “chuangshangxingyingjizhangai” OR “chuangshangyingjizhangai” OR “chuangshanghouyingji” OR “chuangshanghouyingjizhengzhuang” OR “chuangshanghouyingjifanying” OR “PTSD”) AND (“weixianyinsu” OR “yingxiangyinsu” OR “xiangguanyinsu” OR “yuceyinsu” OR “yinsu” OR “yuanyin”). English search terms were (“Myocardial Infarction*” OR “Infarction*, Myocardial” OR “Cardiovascular Stroke*” OR “Stroke*, Cardiovascular” OR “Myocardial Infarct*” OR “Infarct*, Myocardial”) AND (“Stress Disorder*, Post-Traumatic” OR “Stress Disorder, Post Traumatic” OR “Stress Disorder*, Posttraumatic” OR “Post-Traumatic Stress Disorder*” OR “Post Traumatic Stress Disorder*” OR “Posttraumatic Stress Disorder*” OR “Post-Traumatic Neuroses” OR “Posttraumatic Neuroses” OR “Neuroses, Post-Traumatic” OR “Neuroses, Post Traumatic” OR “Neuroses, Posttraumatic” OR “Delayed Onset Post-Traumatic Stress Disorder” OR “Delayed Onset Post Traumatic Stress Disorder” OR “PTSD” OR “Post-Traumatic Stress Symptom*” OR “Posttraumatic Stress Symptom*” OR “PTSS”) AND (“risk factor*” OR “influence factor*” OR “related factor*” OR “Correlation”). At the same time, we try to find and include more relevant literatures through manual retrieval and literature tracking.

### 2.3. Eligibility and exclusion criteria

Inclusion criteria: (1) The subjects were patients diagnosed with myocardial infarction by clinical examination; (2) risk factors or influencing factors of PTSD; (3) at least 1 PTSD assessment tool was used to assess PTSD symptoms; (4) Study types include cohort study, case-control study, and cross-sectional study; published in both Chinese and English, the publication time is the database construction until July 2, 2023. The exclusion criteria were as follows: (1) studies with repeated reports; (2) the subjects had PTSD before the diagnosis of myocardial infarction; (3) the full text of the literature cannot be obtained; (4) literature that cannot provide valid data; (5) patients with myocardial infarction accounted for <50% of the total samples in the study.

### 2.4. Data extraction

Two researchers independently screened the studies, initially screened out the articles to be included after browsing the abstract of the title of the article, extracted the data after reading the whole article, and summarized the data through Excel tables. The extraction content included: first author, publication year, sample size, PTSD assessment tool, PTSD assessment time, PTSD incidence, influencing factors, and study type. After reaching a consensus, the study is finally included. If there is no consensus, the third party will decide whether to include the study.

### 2.5. Risk of bias assessment

The Newcastle-Ottawa Quality Assessment Scale ^[5]^ was used to score the quality of the included literatures in the cohort and case-control studies, with 1 to 3 classified as low quality, 4 to 6 as medium quality, and 7 to 9 as high quality. Cross-sectional studies were scored using the American Agency for Health Care Quality and Research^[6]^ cross-sectional study quality evaluation criteria, with 0 to 3 classified as low-quality literature, 4 to 7 as medium-quality literature, and 8 to 11 as high-quality literature. After reaching a consensus, the study is finally included. If there is no consensus, the third party will decide whether to include the study.

### 2.6. Statistical analysis

Stata17.0 software was used to complete the corresponding statistical analysis. In this study, the combined effect size of the prevalence of PTSD in patients with myocardial infarction was expressed by ES (95% confidence intervals [CI]), and the combined effect size of its influencing factors was expressed by OR (95% CI). The corresponding test was completed by Z-test. If *P* < .05, the combined effect size was statistically significant. The inter-study heterogeneity was assessed by I^2^ and Q tests. If I^2^ is <50% and *P* > .05, it is considered that there is homogeneity among multiple studies, and fixed effect model is adopted. Instead, the random-effects model is used. The sensitivity analysis of prevalence rate was carried out by one-by-one elimination method, and the sensitivity analysis of influencing factors was carried out by comparing the consistency of the results of fixed effect model and random effect model. If the results are inconsistent, it is necessary to remove the literature that has a greater impact on the results and conduct the analysis again. Publication bias is determined by Egger and Begg tests. If *P* > .05, there is no publication bias. For risk factors that could not be integrated in meta-analysis, descriptive integration was used.

## 3. Results

### 3.1. Study selection

**Figure 1:**
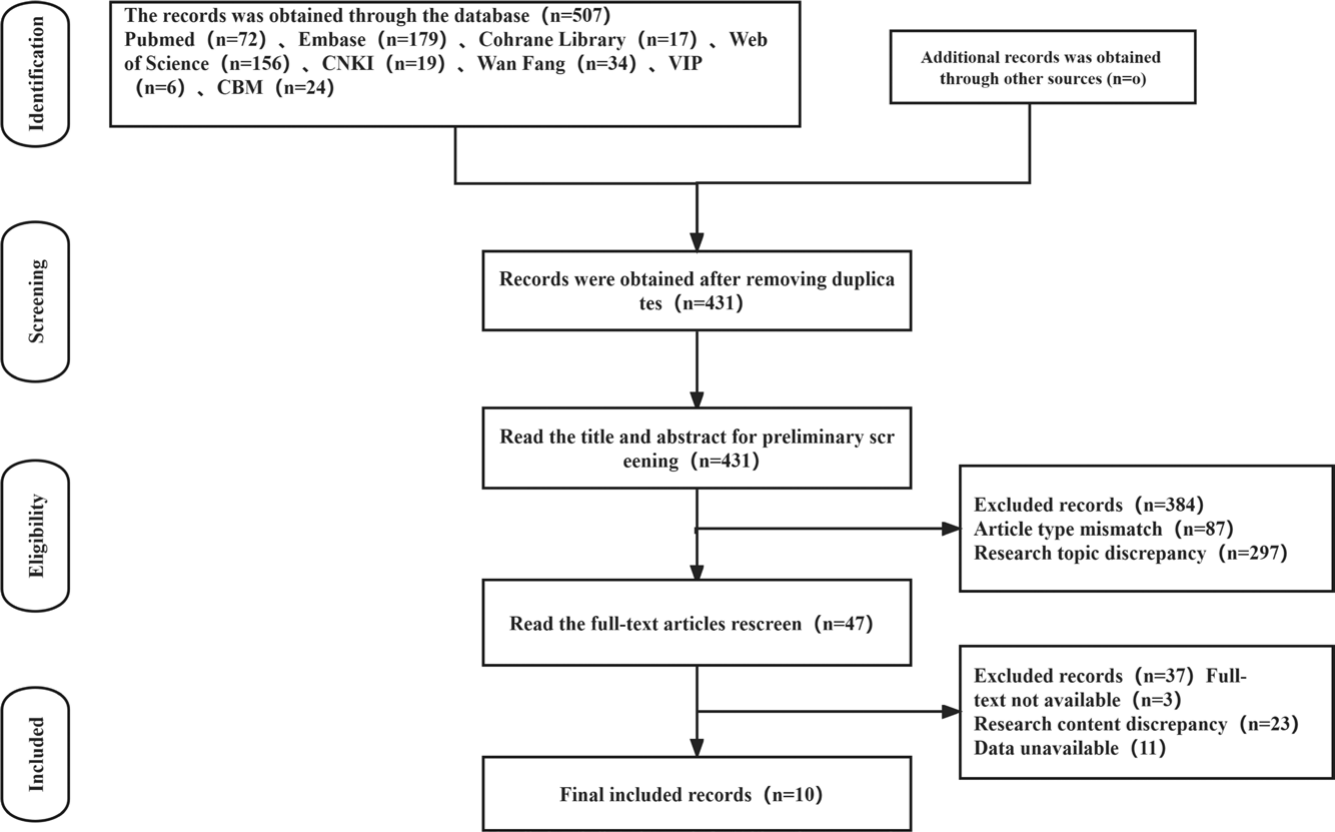
Literature screening flow chart.

A total of 10 literatures were included. Five hundred seven relevant literatures were initially detected in the database, and 76 literatures that were repeatedly published were excluded. After reading the title and abstract, 384 literatures with inconsistent research themes and article types were excluded, 47 literatures were screened out, 37 literatures were rescreened for full text reading, and 10 literatures were finally included (Fig. 1).

### 3.2. Basic characteristics and quality evaluation of the included documents

Ten literatures were included, with a total sample size of 2371 cases. Twenty-six influencing factors such as age, gender, and neuroticism score were extracted. The 10 articles included 2 cohort studies,^[7,8]^ 7 cross-sectional studies,^[9–15]^ and 1 case-control study,^[16]^ published between 2003 and 2022. Assessment tools of PTSD were different in each study, including 4 assessment tools. According to the literature quality evaluation criteria, the quality evaluation of 10 literatures was ≥6 points, and the quality of the included literatures was relatively reliable. The basic characteristics and methodological quality evaluation of the literature are shown in Table 1.

**Table 1:** The basic characteristics and methodological quality evaluation of the literature.

Author	Year	Population	Case	Prevalence rate (%)	PTSD assessment tool	PTSD assessment time	Influencing factor	Research type	Quality score
Bielas^[10]^	2018	183	14	7.7	PDS	3 mo after myocardial infarction	1, 2, 3	Cross-sectional study	8
Cui^[11]^	2022	287	92	32.06	PCL-C	30 d after discharge	4, 5, 6, 7, 8	Cross-sectional study	8
Cao^[12]^	2021	113	23	20.4	PCL-C	3 mo after myocardial infarction	9	Cross-sectional study	9
Pedersen^[7]^	2003	227	33	–	PDS	4–6 wks after myocardial infarction	10, 11, 12	Cohort study	7
Dinenberg^[9]^	2014	579	37	6.4	CDIS	5 y after myocardial infarction	13, 14, 15, 16	Cross-sectional study	8
Pedersen^[8]^	2016	226	34	–	PDS	4–6 wks after myocardial infarction	17, 18, 11	Cohort study	7
Gao^[13]^	2019	266	85	32.0	PCL-C	60 d after myocardial infarction	19, 11, 20	Cross-sectional study	9
Xiong^[14]^	2014	240	68	28.3	PCL-C	More than 1 mo after myocardial infarction	4, 21, 22	Cross-sectional study	8
Liang^[15]^	2016	178	41	23.03	PCL-C	More than 1 mo after myocardial infarction	19, 23, 24, 11	Cross-sectional study	8
Feng^[16]^	2022	72	32	–	PCL-S	–	1, 25, 26, 21	Case-control study	7

Influencing factors: 1 = educational level, 2 = acute stress disorder, 3 = CRP risk category, 4 = sex, 5 = history of diabetes, 6 = creatine kinase isoenzyme, 7 = insomnia score, 8 = disease fear progression, 9 = smoking, 10 = LVEF ≥ 50%, 11 = myocardial infarction, 12 = neuroticism score, 13 = Type D personality, 14 = Total ISEL score, 15 = ISEL belonging domain score, 16 = ISEL tangible domain score, 17 = anxiety, 18 = depression, 19 = age, 20 = invasive rumination score, 21 = Killip scale, 22 = SSRS score, 23 = despair, 24 = fear of death, 25 = number of interventions, 26 = economic income.

CDIS = DSM-IV computer diagnostic interview schedule, CRP = C-reactive protein, ISEL = Interpersonal Support Evaluation List, LVEF = Left Ventricular Ejection Fractions, PCL-C = Posttraumatic Stress Disorder Checklist-Civilian Version, PDS = Posttraumatic Diagnostic Scale, SSRS = Social Support Rating Scale.

### 3.3. Meta-analysis of PTSD prevalence in patients with myocardial infarction

#### 3.3.1. Overall prevalence.

**Figure 2:**
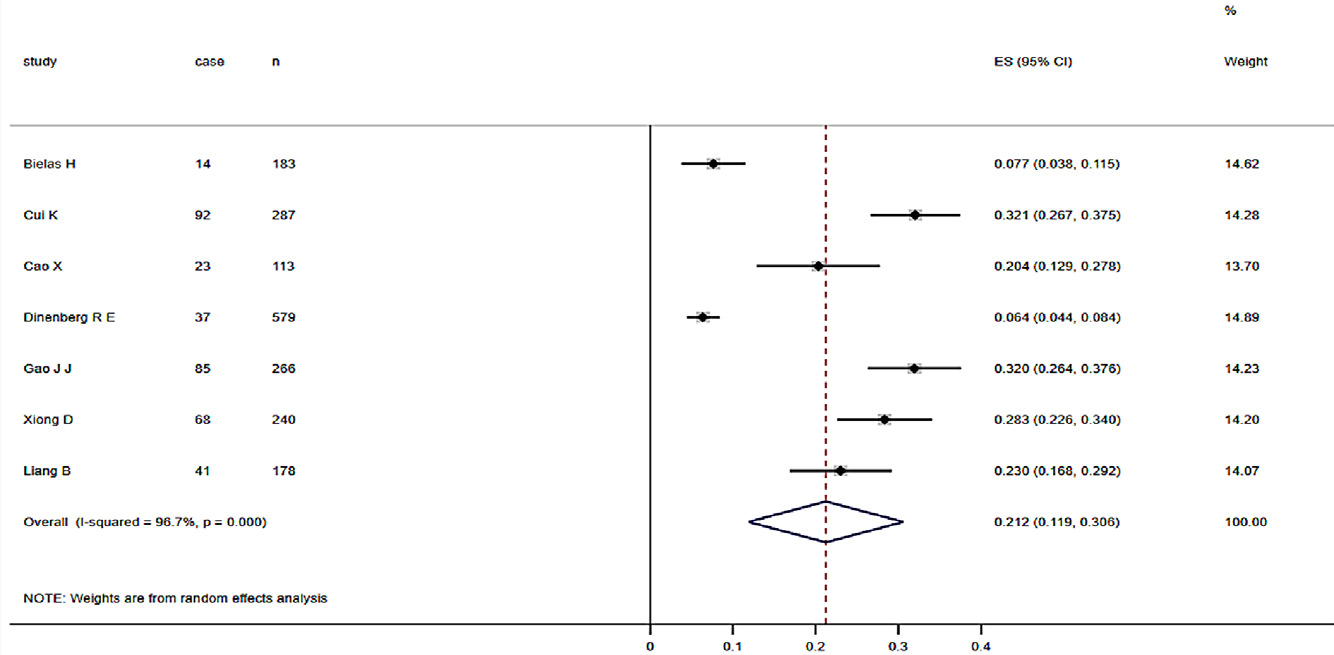
Meta-analysis of PTSD prevalence in patients with myocardial infarction forest map. PTSD = posttraumatic stress disorder.

A meta-analysis of prevalence rates was performed for 7 cross-sectional studies included in the literature, and the heterogeneity test result was (I^2^ = 96.7%, *P* < .001). The random-effects model was used to merge the studies. The results showed that the prevalence of PTSD in patients with myocardial infarction was 21.2% (95% CI, 11.9-30.6), as shown in Figure 2.

#### 3.3.2. Subgroup analysis.

As shown in Table 2, the included studies were subgroup analyzed by study location and diagnostic criteria. According to the study site grouping, China and other countries in patients with myocardial infarction patients with PTSD prevalence was 27.5% (95% CI, 23.1-31.9) and 6.7% (95% CI, 4.9-8.4); group by assessment tool: the prevalence of PTSD in patients with myocardial infarction measured by PDS was 7.7% (95% CI, 3.8-11.5), the prevalence of PTSD in patients with myocardial infarction measured by PCL-C was 27.5% (95% CI, 23.1-31.9), and the prevalence of PTSD in patients with myocardial infarction measured by CDIS was 6.4% (95% CI, 4.4-8.4).

**Table 2:** Subgroup analysis of PTSD prevalence in patients with myocardial infarction.

Analysis item	Number of articles	Population	Case	Heterogeneity test		Meta-analysis results		
				I^2^ (%)	P	Prevalence rate (%)	95% CI	P
Region								<0.001
China	5	1084	309	62.4	0.031	27.5	(23.1, 31.9)	
Other countries	2	762	51	0.0	0.569	6.7	(4.9, 8.4)	
Assessment tools								<0.001
PDS	1	183	14	–	–	7.7	(3.8, 11.5)	
PCL-C	5	1084	309	62.4	0.031	27.5	(23.1, 31.9)	
CDIS	1	579	37	–	–	6.4	(4.4, 8.4)	

CDIS = DSM-IV computer diagnostic interview schedule, PCL-C = Posttraumatic Stress Disorder Checklist-Civilian Version, PDS = Posttraumatic Diagnostic Scale.

#### 3.3.3. Sensitivity analysis.

**Figure 3:**
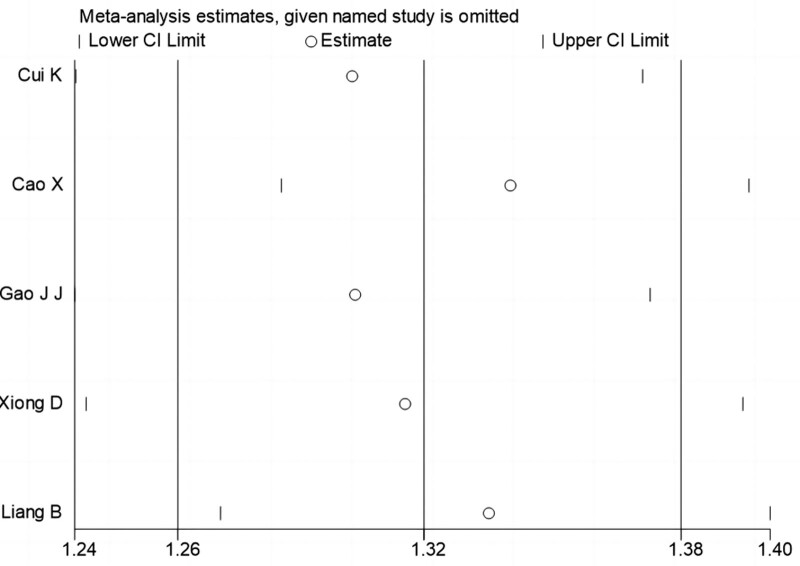
Susceptibility analysis of PTSD prevalence in Chinese patients with myocardial infarction. PTSD = posttraumatic stress disorder.

**Figure 4:**
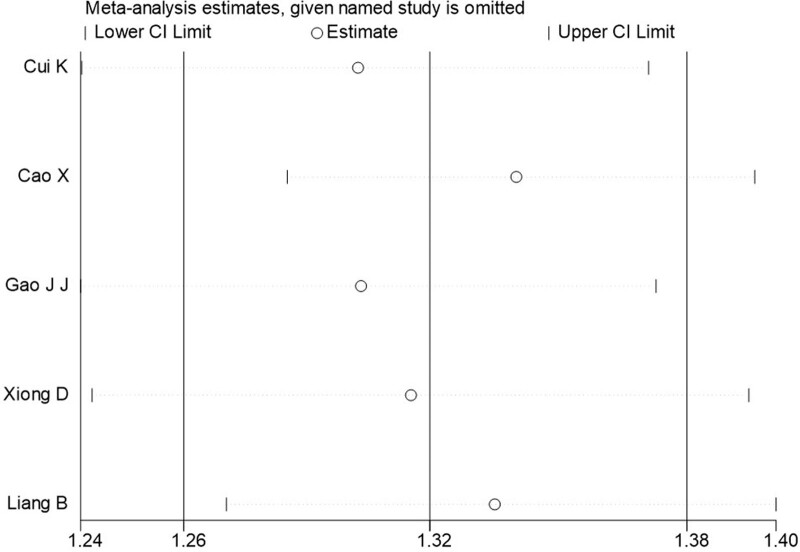
Sensitivity analysis of PCL-C detection of PTSD prevalence in patients with myocardial infarction. PCL-C = Posttraumatic Stress Disorder Checklist-Civilian Version, PTSD = posttraumatic stress disorder.

The sensitivity analysis of the combined results of I^2^ > 50% and >2 included papers was carried out by one-by-one elimination method, and the results showed that the study results were stable. The sensitivity analysis is shown in Figures 3 and 4.

### 3.4. Results of meta-analysis of influencing factors of PTSD in patients with myocardial infarction

#### 3.4.1. Influencing factor.

A meta-analysis of influencing factors was performed for 10 studies included in the literature (Table 3). The results of meta-analysis showed that neuroticism (OR = 2.069) and female (OR = 3.124) were risk factors for PTSD in patients with myocardial infarction, and old age (OR = 0.913) were protective factors for PTSD in patients with myocardial infarction.

**Table 3:** Meta-analysis of influencing factors of PTSD in patients with myocardial infarction.

Influencing factor	Number of articles	Heterogeneity		Model	Combined effect size		P	Publication bias	
		I^2^(%)	P		OR	95% CI		Egger test(P)	Begg tset(P)
Neuroticism score^[7,8,13,15]^	4	98.2	<0.001	Random-effects model	2.069	(1.069,4.006)	0.031	0.907	1.000
Educational level^[10,16]^	2	83.4	0.014	Random effect model	0.547	(1.069,4.484)	0.574	–	1.000
Sex^[11,14]^	2	0.0	0.842	Fixed effect model	3.124	(1.966,4.965)	<0.001	–	1.000
Killip grading^[14,16]^	2	87.1	0.005	Random effect model	2.292	(0.906,5.798)	0.08	–	1.000
Age^[13,15]^	2	0.0	0.602	Fixed effect model	0.913	(0.866,0.961)	0.001	–	1.000

OR = odd ratio

#### 3.4.2. Publication bias.

The Egger test and Begg test results indicate that *P* > .05 for all influencing factors, indicating that the possibility of publication bias among studies is small (Table 3).

#### 3.4.3. Sensitivity analysis.

The sensitivity of the combined results was analyzed by the conversion effect model. As shown in Table 4, the combined results of other influencing factors were stable except Killip classification.

**Table 4:** Sensitivity analysis of influencing factors of PTSD in patients with myocardial infarction.

Influencing factor	Random-effects model	P	Fixed effect model	P
Neuroticism score	2.069 (1.069,4.006)	0.031	2.037 (1.187,2.207)	<0.001
Educational level	0.547 (1.069,4.484)	0.574	1.147 (0.698,1.884)	0.589
Sex	3.124 (1.966,4.965)	<0.001	3.124 (1.966,4.965)	<0.001
Killip grading	2.292 (0.906,5.798)	0.08	2.597 (1.890,3.570)	<0.001
Age	0.913 (0.866,0.961)	0.001	0.913 (0.866,0.961)	0.001

#### 3.4.4. Descriptive analysis.

Descriptive analysis of influencing factors reported in only a single literature, Among them, acute stress disorder,^[10]^ CRP risk category,^[10]^ diabetes history,^[11]^ creatine kinase isoenzyme,^[11]^ insomnia score,^[11]^ disease fear progression,^[11]^ smoking,^[12]^ LVEF ≥ 50%,^[12]^ myocardial infarction,^[7]^ type D personality,^[7]^ interpersonal support (total Interpersonal Support Evaluation List [ISEL] score,^[9]^ ISEL belonging domain score,^[9]^ ISEL tangible domain score^[9]^), anxiety,^[9]^ depression,^[9]^ invasive rumination score,^[13]^ SSRS score,^[14]^ despair,^[15]^ fear of death,^[15]^ number of interventions,^[16]^ economic income,^[16]^ and so on may be a risk factor for PTSD in patients with myocardial infarction.

## 4. Discussion

### 4.1. Prevalence rate

The results of this study showed that the prevalence of PTSD in patients with myocardial infarction was 21.2%, and the subgroup analysis showed that the prevalence of PTSD in patients with myocardial infarction was higher in China than in other countries, which may be related to the different levels of economic and social development, living habits, and public reception of health information among countries. In addition, the prevalence of PTSD in myocardial infarction patients using the PCL-C scale was higher than that of the PDS and CDIS scales. At present, the best assessment tool for PTSD in patients with myocardial infarction needs further research, and studies^[17]^ have shown that the detection rate of PTSD screening tools is higher than that of PTSD diagnosis tools. In the case of the best assessment tool is still uncertain, some researchers use both screening tools and diagnostic tools to test the relevant population, which may improve the effectiveness of the test to some extent. It is worth noting that the Chinese subgroup and the PCL-C subgroup included the same studies, but the results showed high heterogeneity. Although these studies come from the same region and use the same assessment tools, they all have different criteria for determining positive PTSD. For example, Cui’s^[11]^ study takes scale score > 38 as the criterion for determining positive PTSD, while Cao’s^[12]^ study takes scale score ≥ 44 as the criterion for determining positive PTSD. The difference of positive criteria for PTSD may be the source of the high heterogeneity between the Chinese subgroup and the PCL-C subgroup.

### 4.2. Identifying female at-risk groups

This study found that gender is a risk factor for PTSD in patients with myocardial infarction, and female patients are more likely to develop PTSD than male patients after myocardial infarction. Studies have shown a significant correlation between the size of the amygdala and posttraumatic stress disorder when individuals are stressed.^[18]^ Women’s amygdala responds more sensitively and persistently to negative stimuli than men’s, making them more prone to intense emotions such as fear.^[19]^ In addition, due to the difference in sex hormones between men and women, women are more sensitive to painful stimuli, and it is easier to perceive the physical and mental damage caused by diseases than men.^[20]^ Studies have shown that women are twice as likely as men to develop posttraumatic stress disorder.^[21]^ Gender, as an unchangeable factor, requires healthcare professionals to pay more attention to the psychological problems that may arise in female myocardial infarction patients in their clinical work and to actively take effective measures to eliminate the negative emotions of female patients, so as to reduce the risk of PTSD in female myocardial infarction patients.

### 4.3. Valuing the assessment of personality traits

This study found that neuroticism is a risk factor for PTSD in patients with myocardial infarction, which is consistent with the research conclusion of Chung et al,^[22]^ that is, neuroticism is positively correlated with PTSD symptoms. Neurotic people are more likely to react emotionally to negative environmental stimuli, and anxiety and tension are the characteristics of neurotic people.^[23]^ When facing more serious physical diseases, such people are more likely to have strong anxiety, anxiety, and tension, and are prone to misperception, mistaking the disease as a “terminal disease” or a disease that is difficult to cure, mistaking the residual discomfort and discomfort and other physical and mental changes common in normal patients after treatment as pathological or obvious abnormalities, and attempting to remove these physical and mental changes. The more you try to remove it, the worse the symptoms become, creating a vicious cycle that eventually leads to PTSD. Therefore, clinically, for patients with obvious anxiety and tension, their personality characteristics should be screened through the scale if conditions permit, and targeted psychological counseling and humanistic care should be given in time to reduce the risk of PTSD.

### 4.4. Focus on the mental health of young patients

This study found that age was a protective factor for PTSD in patients with myocardial infarction. This conclusion is consistent with the results of studies on the influencing factors of PTSD among African American female HIV patients.^[24]^ The older the age, the lower the risk of PTSD, suggesting that clinical attention should be paid to the mental health of young patients. In the public perception, coronary heart disease such as myocardial infarction is a “proprietary disease” of elderly patients. When young patients have sudden myocardial infarction, they are often psychologically difficult to accept and pessimistic about the prognosis of the disease, so the stress response is more intense. Therefore, in addition to the cardiac function and prognosis of elderly patients, more attention should be paid to the mental health of young patients after myocardial infarction.

### 4.5. Limitations

The sample size of a single study included in this study is limited, and the reliability and stability of the conclusions are limited to some extent. Only Chinese and English literatures were included, and there was a lack of tracking of non-Chinese and English literatures, which may lead to selection bias. Due to the differences in assessment tools and criteria, the combined results of PTSD prevalence in AMI patients need to be treated cautiously, and the combined results of Killip classification are unstable after sensitivity analysis, so the results should be treated with caution. Smoking, diabetes history, economic income, and other influencing factors cannot be meta-analyzed due to insufficient literature, which needs to be verified by large sample and multicenter studies in the future.

### 4.6. Implications

From the results of this study, we can see that regions, assessment tools and diagnostic criteria may be important factors leading to differences in the prevalence of PTSD in patients with AMI. In the future research, we need to further explore the diagnostic criteria and evaluation tools of PTSD, in order to seek the maximum convergence of evaluation tools and diagnostic criteria in the context of regional and cultural differences. It is recommended to conduct a rigorous audit and evaluation of PTSD assessment tools and criteria under the promotion of professional organizations or institutions in order to form recommendations for relevant standards or tools. This may be an effective way to promote the standardization and standardization of PTSD-related research reports, and it can also facilitate the global communication and mutual trust of the research results on the subject. In addition, the results of this study also pointed out that female, neurotic personality and young age were the risk factors of PTSD in patients with AMI. From the perspective of clinical treatment, women, neurotic personality, and young patients should be the focus of attention. When medical resources permit, it is recommended to screen all AMI patients with PTSD and follow-up screening. In view of the sensitive and irritable characteristics of 3 types of patients, such as women, in the course of treatment, we should try our best to keep the hospital environment warm and comfortable, and remove unnecessary instruments and equipment or first aid materials in time, so as to avoid making patients in a state of high stress for a long time. Communication with patients and their families should also be strengthened to help them obtain adequate family support and social support. Cognitive behavioral therapy can be given to patients with negative physical and mental reactions to help patients alleviate acute PTSD symptoms or prevent them from developing into PTSD in the future. Antianxiety, antidepressant, or antipsychotic drugs with serotonin function can be given to patients who cannot be improved by psychotherapy to reduce the psychiatric concomitant symptoms of AMI patients. From the perspective of academic research, first of all, we need more high-quality studies to reflect the epidemiological situation of PTSD in AMI patients in various countries or regions; second, researchers should try to actively explore the prevention and management measures of PTSD in AMI patients on the basis of existing studies, perhaps they can be included in the scope of cardiac rehabilitation programs as a supplement.

## 5. Conclusions

The results of this study show that the prevalence of PTSD in Chinese patients with myocardial infarction is higher than that in other countries, female and neurotic personality are risk factors for PTSD in patients with myocardial infarction, and old age is a protective factor for PTSD in patients with myocardial infarction. Clinical medical staff can refer to the results of this study, strengthen the assessment and screening of PTSD in patients with myocardial infarction, and do a good job in the prevention and management of high-risk patients, so as to reduce the incidence of PTSD.

## Author contributions

**Conceptualization:** Jingyu Liu, Lingyu Wang.

**Data curation:** Jingyu Liu.

**Formal analysis:** Jingyu Liu.

**Investigation:** Jingyu Liu.

**Methodology:** Jingyu Liu, Lingyu Wang.

**Software:** Yimu Wang.

**Writing – original draft:** Jingyu Liu, Lingyu Wang.

**Writing – review & editing:** Haiyan Fang, Xiang Wang.
